# Antagonists Enhance Cell-Surface Expression of Mammalian Odorant Receptors

**DOI:** 10.3390/ijms26041458

**Published:** 2025-02-10

**Authors:** Ikumi Takayama, Nako Araki, Jeevan Tewari, Masafumi Yohda, Hiroaki Matsunami, Yosuke Fukutani

**Affiliations:** 1 Department of Biotechnology and Life Science, Tokyo University of Agriculture and Technology, Koganei 184-8588, Tokyo, Japan; ikumi.takayama@yohda.net (I.T.); nako.araki@yohda.net (N.A.); yohda@cc.tuat.ac.jp (M.Y.); 2 Department of Molecular Genetics and Microbiology, Duke University Medical Center, Durham, NC 27710, USA

**Keywords:** GPCR, odorant receptor, antagonist, chaperone

## Abstract

Functional characterization of vertebrate odorant receptors (ORs), members of the G protein-coupled receptor (GPCR) family, is essential for understanding olfaction. However, the functional expression of ORs in heterologous cells is often challenging, at least partly caused by structural instability in non-olfactory cells. Antagonists have been shown to restore membrane expression of some non-olfactory GPCR mutants, likely by transient increase in structural stability upon antagonist binding. Based on this premise, we examined whether antagonists could enhance OR membrane expression in heterologous cells. Using phenyl salicylate (PES) on cells expressing the mouse OR Or11g7, we observed increased cell surface expression exceeding the effects of co-expression with the OR chaperone RTP1S. After removing the antagonist, Or11g7 retained normal agonist responsiveness. Similar enhancements in cell surface expression were observed for a human OR OR2T11 treated with its antagonists. These findings suggest that small-molecule antagonists act as pharmacological chaperones to stabilize OR conformation, enhancing surface expression in a manner similar to molecular chaperones. Our study reveals a novel role for odorant antagonists in OR biogenesis and may inform future research on olfactory training mechanisms.

## 1. Introduction

Organisms have developed the capacity to detect environmental chemicals, a sensory ability termed olfaction in animals, which is crucial for detecting a wide array of odor molecules. The olfactory process begins as volatile substances enter the nasal cavity and bind to odorant receptors (ORs) located on the membrane of the olfactory sensory neurons in the olfactory epithelium [1]. ORs are a part of the Class A G protein-coupled receptor (GPCR) family in vertebrates, the most expansive receptor family, with humans possessing about 350 types and mice around 1100 [2,3,4]. Notably, each mature olfactory sensory neuron expresses a singular type of OR, a phenomenon known as the “one neuron–one OR” rule despite specific regional expression of ORs in the olfactory epithelium [5,6]. Odor discrimination depends on combinatorial coding, where diverse OR responses enable the detection and differentiation of environmental odors [7,8,9,10].

ORs have uniquely evolved to identify various odor molecules, but their vast diversity might undermine their structural integrity, making them prone to less effective expression in heterologous cell systems compared to other GPCRs. OR proteins are highly abundant at the cilia membranes in vivo [11,12,13]. However, in heterologous systems such as HEK293T cells and other commonly used cell lines [14,15,16,17,18,19,20], the expression levels of the vast majority of ORs are significantly lower [21]. Typically, most ORs in such systems are retained in the endoplasmic reticulum through its quality control mechanisms and tagged for degradation [22]. Over the past >25 years, numerous strategies have been developed by many labs to enhance OR membrane expression in heterologous cells, aiming to achieve more physiologically relevant receptor expression conditions [23]. One such approach is the co-expression of chaperones such as receptor transporting proteins RTP1S and RTP2 [24,25,26], Ric-8b [27,28], and heat shock protein HSP70 [29,30]. Another strategy involves attaching tags that foster membrane association, like Rho- or Lucy-tags, to ORs [31,32]. Additionally, creating ORs with stabilizing mutations or consensus sequences that align with highly conserved amino acid sequences across orthologs can also promote better cell-surface membrane localization, although such changes may modify their agonist specificity [33,34,35]. Given that many ORs are still resistant to functional expression in heterologous cells, there is a clear need for novel methods to improve OR expression in these systems.

An effective strategy for increasing the cell membrane localization of “difficult to express” GPCRs may include utilizing small molecules that bind to these receptors. For instance, the mutant V2 vasopressin receptor, V2R del 62-66, displays significantly lower cell surface expression compared to its wild-type counterpart. However, when the antagonist SR121463A is introduced, there is a notable increase in the surface expression of V2R del 62-66, along with a recovery in its agonist responses. This suggests that antagonists can enhance the correct folding and maturation of mutant GPCRs, improving their functionality and surface expression [36]. It has also been reported that small molecules, acting as agonists or antagonists for β1-adrenergic receptors [37] and A1-adenosine receptors [38], function as pharmacological chaperones to assist in the cell surface expression of these receptors. In this way, it has been demonstrated that small molecules can influence protein folding and transport to the cell membrane in several GPCRs other than ORs, and that small molecules functioning as pharmacological chaperones exist.

Based on these insights, we hypothesized that the interaction of ORs with small molecule antagonists inside the cell may temporarily boost their structural stability and enhance their cell surface membrane presence. Antagonist chaperones are likely to be more desirable than agonist chaperones due to the potential for diminished or abolished cellular responses in the presence of agonists, resulting from desensitization, adaptation or cellular exhaustion associated with prolonged signaling. In this study, we investigated whether the addition of antagonists can improve the cell membrane expression of well-characterized ORs with known antagonists.

## 2. Results and Discussions

### 2.1. Enhanced Surface Membrane Localization of Or11g7 by Its Antagonist, Phenethyl Salicylate

**Figure 1:**
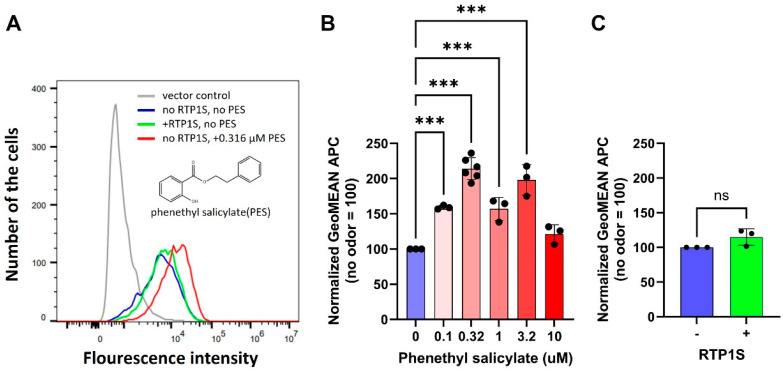
PES enhances the cell-surface expression of Or11g7. (**A**) Cell surface expression of Or11g7 in HEK293T cells is enhanced by the addition of phenyl salicylate (PES). Blue: Or11g7 alone, green: +RTP1S, Red: +0.32 µM PES, gray: vector control. The cell surface expression level was quantified via APC fluorescence intensity (X-axis) against cell number (Y-axis). (**B**) Dose response in Or11g7 cell surface expression analysis. The APC geometric values for Or11g7 without PES supplementation were normalized to 100. Error bars represent the standard deviation (SD) for n = 3. Statistical analysis involved one-way ANOVA (F(5, 12) = 39.03, *p* < 0.001) followed by Dunnett’s test (*** *p* < 0.001). (**C**) RTP1S co-expression does not enhance the Or11g7 expression in HEK293T cells. Comparisons between conditions with and without RTP1S co-expression were conducted using a two-sided Student’s *t*-test (non-significant (ns), *p* > 0.05).

There are currently only a few known antagonists for specific ORs. When investigating the enhancement of cell membrane expression by antagonists, we considered it effective to use ORs with relatively low membrane expression and known antagonists. For our study, we primarily focused on the combination of Or11g7, also known as Olfr740 and MOR106-4, and phenethyl salicylate (PES) [39]. After transfecting HEK293T cells with an Or11g7 expression vector, we immediately supplemented the culture medium with PES. The cell-surface localization of Or11g7 was assessed using fluorescent-activated cell sorting (FACS) with an antibody against the N-terminal rhodopsin tag. ORs are inherently hydrophobic, resulting in poor membrane transfer efficiency during blotting and a tendency to aggregate in SDS-containing buffers used for sample preparation and gel electrophoresis [40,41,42]. Given that the primary objective of this study was to determine whether the presence of an antagonist enhances the cell surface expression of ORs, we selected FACS as the most effective method for quantifying surface expression levels. The results indicated that the presence of PES in the culture medium significantly increased the membrane expression of Or11g7 (Figure 1A).

A concentration-dependent analysis, performed using ligand concentrations incremented in 0.5-log intervals, revealed the greatest enhancement in membrane expression at 0.32 μM (log(PES), M = −6.5), suggesting that approximately 0.32 μM is the optimal concentration for increasing the membrane localization of Or11g7. Contrary, surface expression of Or55b4, also known as Olfr544 and OR-S6, which does not respond to PES as either an agonist or an antagonist, was not enhanced significantly by addition of PES (Figure S1). Interestingly, higher concentrations of PES were observed to be less effective in enhancing cell surface expression (Figure 1B). To address this, we conducted a cell viability assay (CellTiter-Glo) on cells treated with PES, which indicated that PES does not significantly impact cell proliferation (Figure S2). Additionally, a slight but significant reduction in total Or11g7 protein levels upon PES addition was observed, suggesting that the increased surface expression of Or11g7 is not due to higher total protein levels (Figure S3). Instead, this supports our hypothesis that PES enhances Or11g7 membrane expression by facilitating its transport to the plasma membrane and/or stabilizing it on the cell surface. Furthermore, it is possible that concentrations of PES at 10 μM or higher may affect the intracellular protein synthesis or trafficking systems. The effect of co-expression with the OR chaperone RTP1S showed only a marginal increase in the surface expression of Or11g7 (Figure 1C). Enhancement of surface expression of Or11g7 by PES supplementation was also observed in the RTP1S co-expression condition (Figure S4). Based on our extensive experience with numerous human and mouse ORs, those exhibiting moderate levels of expression generally show increased expression with RTP1S [7,10,24,26,35,43]. Therefore, the specific membrane expression behavior of Or11g7 appears to be a rare exception. The enhancement of membrane localization by PES was greater than that achieved through co-expression with RTP1S, highlighting the superior role of the antagonist in enhancing Or11g7 expression.

**Figure 2:**
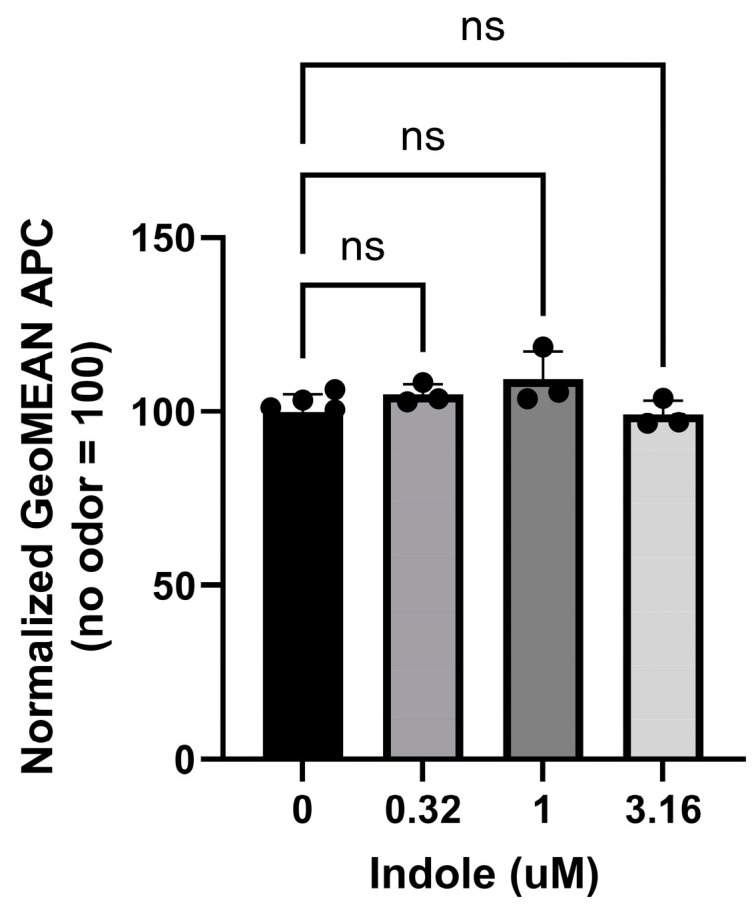
Effect of indole on membrane expression of Or11g7. Cell surface expression levels of Or11g7 after the addition of indole (agonist). The APC geometric mean values for Or11g7 without odor supplementation were normalized to 100. Error bars represent the standard deviation (SD) for n = 3. Statistical analysis was performed using one-way ANOVA followed by Dunnett’s test (non-significant (ns), *p* > 0.05).

Furthermore, we investigated the effect on membrane expression using indole, an agonist of Or11g7. A slight increased tendency in membrane expression was observed in the presence of 1 µM indole; however, the effect was not statistically significant (Figure 2).

To explore binding sites of Or11g7 to PES, we utilized AlphaFold2 to generate model structures and performed docking simulations of ligands using AutoDock [44,45]. In our model, Or11g7 forms hydrogen bonds at S113 and hydrophobic interactions at F108, F109, Y263, and Y282, which serve as common binding sites for both indole and PES (Figure S5). PES is predicted to interact with more binding sites on the OR compared to indole, suggesting that the overall conformational changes induced by PES and indole may differ. Additionally, we examined the effect of co-administration of the agonist indole and the antagonist PES on the expression of Or11g7. Indole addition inhibited the PES-induced enhancement of Or11g7 membrane localization (Figure S6). This finding supports our hypothesis that PES’s effect is specifically dependent on its binding to Or11g7. However, the possibility that PES binds to an allosteric site cannot be entirely excluded. These findings suggest that PES, a hydrophobic molecule, permeates the lipid bilayer and may bind to Or11g7, thereby stabilizing its three-dimensional structure in an inactive form.

What are the potential mechanisms by which antagonists enhance the cell surface expression of ORs? One possibility is that antagonist binding facilitates OR targeting to the cell surface during synthesis. ORs are known to predominantly accumulate in the ER when expressed in heterologous systems such as HEK293T cells [22,25]. This mechanism is consistent with the action of pharmacological chaperones observed in non-olfactory GPCRs, such as V2R and δOR, where it has been shown that non-membrane-permeable ligands do not enhance membrane expression. These findings suggest that the antagonists used in this study primarily function by binding to receptors in their unstable, partially folded states within the ER, stabilizing their structure [46,47]. To gain mechanistic insights into PES-induced Or11g7 membrane localization, we conducted kinetic experiments. We observed a decrease in Or11g7 cell surface expression when PES was added at later time points (Figure S7). This is not consistent with the idea that PES stabilizes Or11g7 on the cell surface by preventing its internalization. Instead, our data support the hypothesis that PES enhances Or11g7 surface expression by promoting its trafficking through improved folding at the ER.

However, alternative or additional mechanisms cannot be excluded as explanations for the observed increase in cell surface expression mediated by antagonists. For example, antagonists may inhibit constitutive receptor internalization or enhance receptor recycling to the plasma membrane [48,49]. Future studies, modeled on approaches used for other GPCRs, such as kinetic experiments using stable cell lines, cell-impermeable antagonists, and fluorescently labeled antagonists, could provide valuable insights into these underlying mechanisms [50,51]. Such advancements may help elucidate receptor trafficking and localization processes in greater detail.

One possible reason why indole, despite its ability to bind to Or11g7, did not exhibit an enhancement effect is that structural differences of ORs with an agonist or an antagonist, potentially affecting trafficking efficiencies. Even if indole temporarily support membrane expression, OR endocytosis mediated by β-arrestin following agonist activation may prevent sustained membrane expression [52]. Another possibility is the adverse effects on cells from prolonged signaling. Further investigation is necessary to test these possibilities.

### 2.2. Surface Membrane Localization of Or11g7 Is Selectively Enhanced by PES, Suggesting the Potential for This Effect to Also Be Observed in an Olfactory Mucus Environment

**Figure 3:**
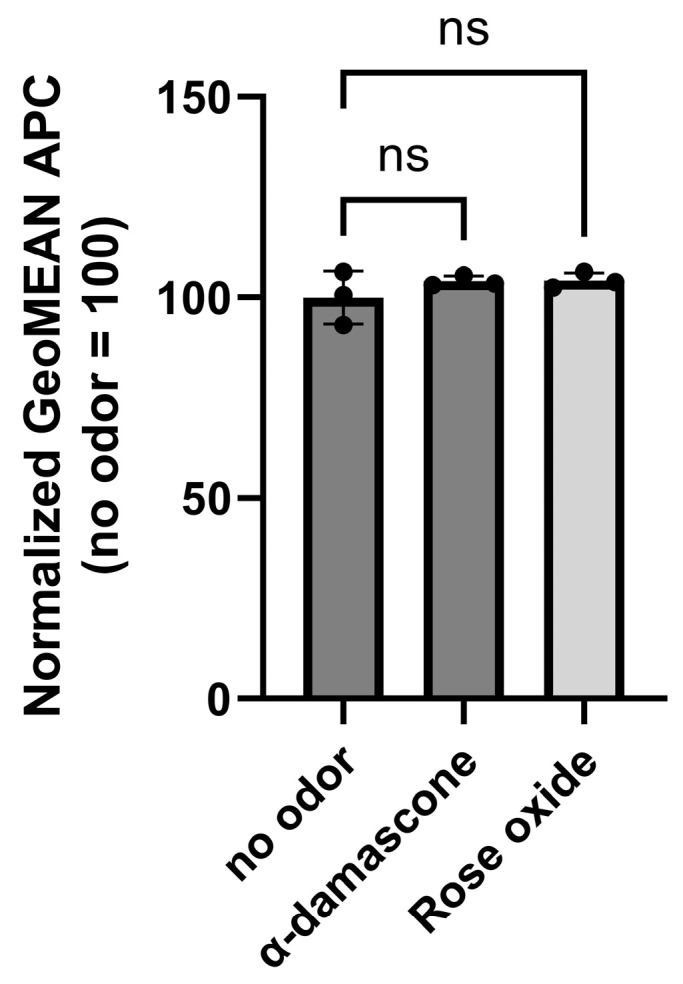
PES selectively enhances membrane expression of Or11g7. Cell surface expression levels of Or11g7 after the addition of alpha-damascone (neither agonist nor antagonist) and rose oxide (neither agonist nor antagonist). The APC geometric mean values for Or11g7 without odor supplementation were normalized to 100. Error bars represent the standard deviation (SD) for n = 3. Statistical analysis was performed using one-way ANOVA followed by Dunnett’s test (non-significant (ns), *p* > 0.05).

If PES binding selectively stabilizes the folding conformation of Or11g7, treatment with other molecules should not affect the cell surface expression of Or11g7. To test this hypothesis, we conduct similar experiments using compounds that are neither agonists nor antagonists of Or11g7 (α-damascone and rose oxide) [39]. The results showed the addition of these odorants did not enhance the membrane expression of Or11g7 (Figure 3).

**Figure 4:**
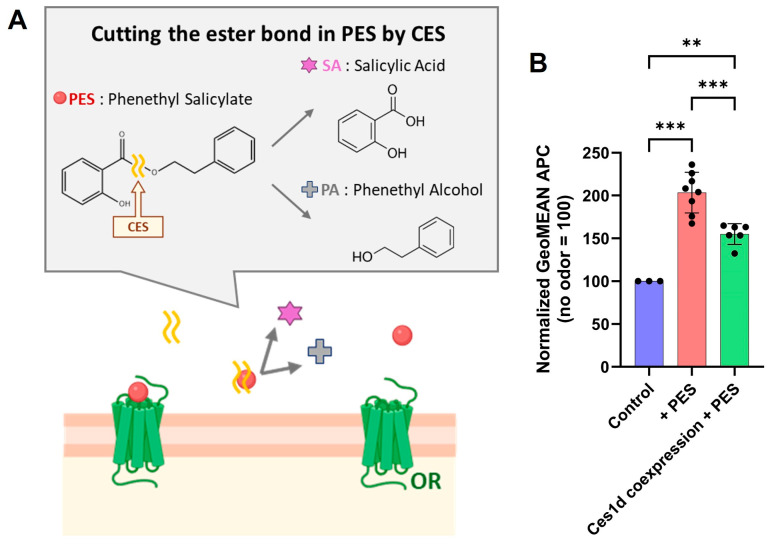
Influence of a metabolic enzyme on the enhancement of Or11g7 expression by antagonists. (**A**) Schematic image of hydrolysis of ester odor by carboxyl esterase outside of cells. Salicylic acid and phenethyl alcohol were produced by enzymatic reaction of secreted carboxyl esterase. (**B**) Relative expression of olfr740 with and without carboxyl esterase (CES1d). Statistical analysis involved one-way ANOVA (F(2, 14) = 37.13, *p* < 0.001) followed by Tukey’s test (** *p* < 0.01, *** *p* < 0.001).

In olfactory epithelium, olfactory sensory neurons expressing ORs are covered by olfactory mucus [10,53]. Carboxylesterases are known to be present in the olfactory mucus, and they are believed to be primarily secreted by Bowman’s glands [54]. Previous studies demonstrated that the eugenol-responsive Olfr979 exhibited a response to eugenol acetate only when co-expressed with Ces1d. Additionally, changes in responses to carboxylic acid esters were observed in other ORs under similar conditions [10]. Since PES could be hydrolyzed by the carboxylesterase after dissolving under these conditions, to mimic the olfactory mucus environment, we tested the influence of co-expression of Ces1d, a major carboxyl esterase expressed in the olfactory epithelium [52], on Or11g7 expression in the presence of PES (Figure 4A). Co-expression of Ces1d led to a reduction in Or11g7 membrane expression compared to cells without Ces1d co-expression (Figure 4B). However, Or11g7 expression in presence of Ces1d was still significantly greater than in the absence of PES, indicating that the membrane expression-enhancing effect of PES was moderately maintained, even under conditions mimicking the olfactory mucus. A limitation of our experimental system is that Ces1d was co-expressed with the ORs, likely resulting in co-expression within the same cells. Nevertheless, Ces1d retained its functionality in this experimental context, supporting its role in facilitating the observed effects.

For future studies, alternative approaches could be explored, such as expressing the metabolic enzyme in separate cells and co-culturing them with OR-expressing cells, or adding purified carboxylesterase to the culture medium. Since various enzymes other than carboxylesterases are expressed in nasal mucus [55,56,57,58], future research could also examine the potential roles of other enzymes in modulating OR function and expression.

### 2.3. Sustained Cell-Surface Expression and Agonist Responsiveness Post-Antagonist Removal

**Figure 5:**
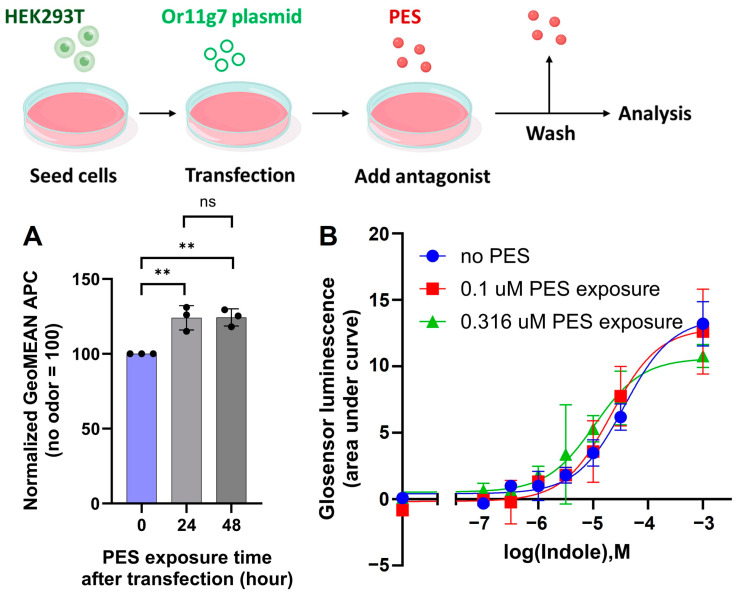
Effect of antagonist washing on cultured Or11g7-expressing cells. (**A**) Comparison of Or11g7 expression in cells cultured for 48 h with and without a medium change, including conditions with and without the antagonist. Error bars represent the standard deviation (SD) for n = 3. Statistical analysis involved one-way ANOVA (F(2, 6) = 17.60, *p* = 0.003) followed by Dunnett’s test (non-significant (ns), *p* > 0.05; ** *p* < 0.01). (**B**) Dose- response of Or11g7-expressing cells to varying concentrations of indole (a known agonist of Or11g7) following culture in medium containing the antagonist and subsequent washing with PBS.

A key question is whether Or11g7, expressed in the presence of its antagonist, can retain its agonist responsiveness after the antagonist is removed. To investigate this, we examined the stability of Or11g7 surface expression after washing out PES. Or11g7-expressing cells were cultured overnight in PES-supplemented medium, then washed with PBS (phosphate-buffered saline) and further cultured in 24 h in PES-free medium. No difference was observed in Or11g7 membrane expression between the washed samples (without PES for the final 24 h) and the unwashed samples (with PES maintained for 48 h) (Figure 5A). This finding indicates that Or11g7, once expressed on the membrane with the assistance of an antagonist, sustains surface expression even after the antagonist’s removal. Additionally, following PES washout, Or11g7 demonstrated an equivalent response to its agonist indole compared to cultures without PES (Figure 5B), confirming that ORs can restore native agonist responsiveness once the antagonist is removed. There are various odor molecules present in the environment, which continuously enter the nasal cavity. Based on this observation, the expression of ORs that respond antagonistically to these environmental odor molecules is likely to be higher in individuals exposed to such odors regularly, compared to those in different environments.

When such individuals move to an environment where these antagonist odor molecules are absent, the inhibitory effect is lifted, potentially leading to the activation of the upregulated ORs. This hypothesis is consistent with reports of environmental factors altering olfaction [52], as well as findings that olfactory training can enhance the perception of odors [59,60,61]. These phenomena suggest that interactions between odor molecules and ORs may influence OR expression, particularly through mechanisms involving small-molecule interactions during protein expression. 

### 2.4. Antagonist-Induced Enhancement of Human OR Expression

**Figure 6:**
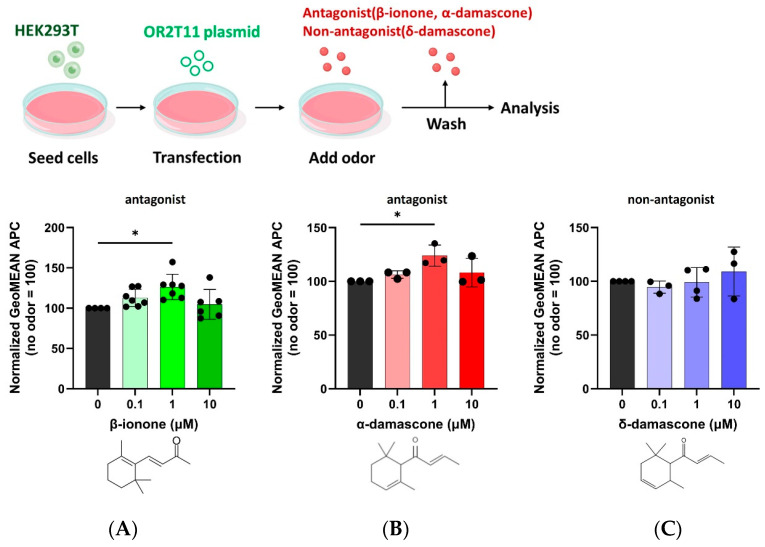
Antagonist concentration-dependent enhancement of cell surface expression in human OR2T11. The APC geometric values for OR2T11 with normal medium without odor supplement were normalized to 100. (**A**) β-ionone (antagonist). Error bars represent the standard deviation (SD) for n = 6. Statistical analysis involved one-way ANOVA (F(3, 22) = 4.012, *p* = 0.02) followed by Dunnett’s test (* *p* < 0.05). (**B**) α-damascone (antagonist). Error bars represent the standard deviation (SD) for n = 3. Statistical analysis involved one-way ANOVA (F(3, 8) = 4.345, *p* = 0.04) followed by Dunnett’s test (* *p* < 0.05). (**C**) δ-damascone (not antagonist). Error bars represent the standard deviation (SD) for n = 3. Statistical analysis involved one-way ANOVA (F(3, 10) = 0.668, *p* = 0.59) followed by Dunnett’s test (*p* > 0.05).

We further investigated whether the antagonist-induced enhancements in OR expression observed in the mouse model OR, Or11g7, could also apply to human ORs. Using known antagonists, α-damascone and β-ionone, which target the thiol-responsive OR2T11, we evaluated changes in OR2T11 surface expression [62]. Both antagonists significantly increased the surface expression of OR2T11 (Figure 6). In contrast, δ-damascone, which lacks antagonist activity, did not enhance the OR2T11 membrane expression, indicating molecular selectivity in the enhancement process. These findings suggest that ORs, especially those with low initial membrane localization, can benefit substantially from the presence of an effective antagonist. Similar to the findings for Or11g7 (Figure 1B), a decrease in the OR membrane expression enhancement effect was also observed for OR2T11 at 10 μM, suggesting that this phenomenon may be common across multiple receptor–antagonist pairings. However, testing this hypothesis remains challenging due to the largely unresolved mechanisms governing the intracellular synthesis and trafficking of ORs to the cell membrane. Further studies are necessary to elucidate these processes and determine the potential impacts of high antagonist concentrations on receptor expression and cellular function.

It is likely that antagonists stabilize and maintain ORs on the cell surface, preventing their degradation. Additionally, antagonists may penetrate the cell and bind to incompletely folded ORs, facilitating proper folding. In this study, we used known antagonists; however, the effects of small molecules may vary based on ligand type and OR membrane expression levels. Further research with molecules that have high membrane permeability and antagonist activity could lead to the discovery of small molecules that significantly enhance OR cell surface expression.

## 3. Materials and Methods

### 3.1. DNA and Vector Preparation

The open reading frames of the mouse Or11g7 and human OR2T11 genes were cloned from the mouse or human genome, respectively, and subcloned into pCI (Promega, Madison, WI, USA) with a rhodopsin tag (Rho-tag) at the N-terminus, which has been previously demonstrated to enhance the plasma membrane localization of multiple ORs [63]. The other expression vectors containing mouse RTP1S, human RTP1S, mouse Ces1d [10] or GloSensor-F22 (Promega, Madison, MI, USA), and AcGFP (Takara Bio, Shiga, Japan) were amplified and purified by NucleoSpin plasmid TF grade (Takara Bio, Shiga, Japan). All plasmid sequences were verified using Sanger sequencing (3100 Genetic Analyzer, Applied Biosystems, Waltham, MA, USA).

### 3.2. Cell Culture

HEK293T cells (purchased from Thermo Fisher Scientific, Waltham, MA, USA) were maintained in Dulbecco’s Modified Eagle Medium (D-MEM) containing 10% FBS (vol/vol) with L-Alanyl-L-Glutamine, penicillin-streptomycin, and amphotericin B at 37 °C and 5% CO_2_. No mycoplasma infection was detected.

### 3.3. Flow Cytometry Analysis (FACS)

HEK293T cells were grown to confluence, resuspended, and seeded onto 35 mm plates at 25% confluency. The cells were cultured overnight. The Rho-tagged ORs in the plasmid pCI and AcGFP-N1 expression vector (Takara Bio, Shiga, Japan) were transfected using FuGENE 4K transfection reagent (Promega, Madison, WI, USA). When RTP1S and Ces1d were coexpressed, the respective plasmid DNAs were mixed during transfection. During transfection, the total amount of plasmid DNA introduced was equalized by adding empty vectors, ensuring consistent experimental conditions. Odor solutions were added to the medium after the transfection reaction at the desired concentration. After 20–22 h, the cells were resuspended in a cell stripper (Corning, New York, NY, USA) and then kept on ice. The cells were spun down at 4 °C and resuspended in PBS containing 2% FBS to wash away the cell strips. They were then incubated with a mouse anti-Rho4D2 as the primary antibody (Merck-Millipore, Burlington, MA, USA), washed, and stained with APC-conjugated goat anti-mouse IgG antibody (BioLegend, San Diego, CA, USA) in the dark. To stain dead cells, 7-amino-actinomycin D (Merck-Millipore, Burlington, MA, USA) was added. The cells were analyzed using a CytoFLEX flow cytometer (Beckman Coulter, Brea, CA, USA) with gating allowing single, spherical, viable, and GFP-positive cells, and the measured APC fluorescence intensities were analyzed and visualized using FlowJo software (V10. 10. 0, Becton, Dickinson and Company, Franklin Lakes, NJ, USA). The FACS analysis experiments were repeated at least three times.

For confirmation of total protein expression of Or11g7, collected cells were fixed with 4% (*w*/*v*) paraformaldehyde for 20 min at room temperature, permeabilized with 0.2% Triton-X100, and immunostained with the same antibody for both primary and secondary antibodies.

### 3.4. GloSensor Analysis

All odorants were purchased from FUJIFILM Wako Chemicals (Osaka, Japan) and TCI Chemicals (Tokyo, Japan). A GloSensor cAMP Assay (Promega, Madison, MI, USA) was used to measure the changes in cAMP levels caused by receptor activation upon ligand binding [64]. HEK293T cells were plated on poly-D-lysine-coated 96-well plates. Eighteen to 24 h after plating, cells were transfected with 80 ng/well of plasmids encoding ORs and 10 ng/well of Glosensor-22F plasmid (Promega, Madison, MI, USA). When odorants were added, they were added to the medium after the transfection reaction. Eighteen to 24 h later, the medium was replaced with 50 μL of HBSS (Gibco) containing 10 mM HEPES and 1 mM glucose, followed by 25 μL of HBSS containing GloSensor cAMP Reagent (Promega, Madison, MI, USA). The plates were kept in the dark at room temperature for two hours to equilibrate the cells with the reagent. The test plate was inserted into the plate reader. The luminescence derived from basal activity in each OR was measured. For liquid stimulation, cells were stimulated by the addition of an odorant diluted in HBSS containing 10 mM HEPES and 1 mM glucose. Soon after odorant stimulation, the test plate was inserted into a GloMax Discover Microplate Reader (Promega, Madison, MI, USA). The luminescence in each well was measured at 60 s intervals for 20 cycles. All luminescence values were divided by the value obtained from cells before odor addition. The AUC values obtained from cells under the same conditions that had been transfected with an empty vector were then subtracted from all AUC values. The GloSensor analysis experiments were conducted in triplicate.

### 3.5. Cell Viability Assay

HEK293T cells were cultured in a 96-well plate for 24 h, and then odorants were added at the desired concentration. After 24 h, the samples from which the odor was to be removed were washed with PBS, medium was added, and incubated for another 24 h. After that, the same amount of CellTiter-Glo^®^ 2.0 Reagent (Promega, Madison, WI, USA) as the medium was added to all samples, and the samples were mixed by shaking for 2 min. After leaving the samples at room temperature for 10 min, luminescence was measured. The values of luminescence were normalized such that the value of each well without PES was defined 1.0.

### 3.6. Statistical Analysis

Multiple comparisons were performed using one-way analysis of variance (ANOVA) followed by Dunnett’s test and Tukey’s test using GraphPad Prism. Unpaired Student’s *t*-tests were performed using GraphPad Prism. The average is shown as the mean ± standard deviation.

## 4. Conclusions

This study highlights the potential of antagonists to stabilize the three-dimensional structure of ORs, providing a promising solution to the prevalent issue of OR retention in the endoplasmic reticulum due to cellular quality control mechanisms during heterologous cell expression. Given the inherently low expression levels of ORs, their maturation and trafficking mechanisms remain poorly understood. Moreover, pharmacological chaperones targeting ORs, which form extensive subfamilies, have not been reported to date. Identifying small molecules that enhance OR membrane expression is therefore of critical importance. Notably, the ability of these molecules to directly stabilize OR structure suggests applications beyond cellular models. For instance, olfactory training, widely employed to improve odor perception in individuals with diminished smell due to viral infections or drug side effects, might benefit from these findings [65,66]. It is plausible that, during daily exposure to training odors, odorants could bind to ORs, promoting their trafficking to the cilia membrane and enhancing olfactory function. These insights are expected to make substantial contributions to various research fields centered on OR biology and function.

## Data Availability

Any additional information required to reanalyze the data reported in this paper is available from the lead contact upon request.

## Supplementary Materials

The following supporting information can be downloaded at: https://www.mdpi.com/article/10.3390/ijms26041458/s1.
