# Supplementary material for: Antagonists Enhance Cell-Surface Expression of Mammalian Odorant Receptors
Source: Int J Mol Sci. 2025 Feb 10;26(4):1458. doi: 10.3390/ijms26041458 (PMC11855683; doi:10.3390/ijms26041458)
Supplement: Supplementary file 1 [file ijms-26-01458-s001.zip › ijms-3390715-supplementary.pdf]

Supplementary information

Title

Antagonist enhances cell-surface expression of mammalian olfactory receptors.

Authors

Ikumi Takayama[1*], Nako Araki[1*], Jeevan Tewari[2], Masafumi Yohda[1], Hiroaki Matsunami[2†] and Yosuke Fukutani[1†]

Affiliations

[1]Department of Biotechnology and Life Science, Tokyo University of Agriculture and Technology, Koganei, Tokyo 184-8588, Japan

[2]Department of Molecular Genetics and Microbiology, Duke University Medical Center, Durham, NC 27710, USA

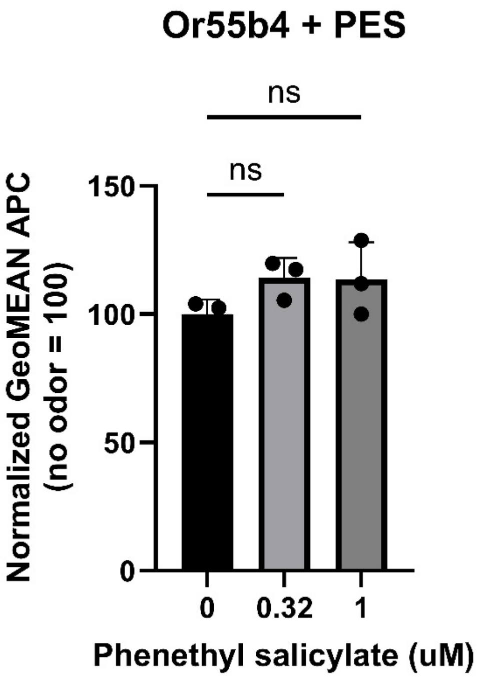

Figure S1 PES does not significantly enhance Or55b4 cell surface expression
Cell surface expression levels of Or55b4 after the addition of Phenyl salicylate (PES), The APC geometric mean values for Or55b4 without odor supplementation were normalized to 100. Error bars represent the standard deviation (SD) for n=3. Statistical analysis was performed using one-way ANOVA followed by Dunnett's test ($p > 0.05$).

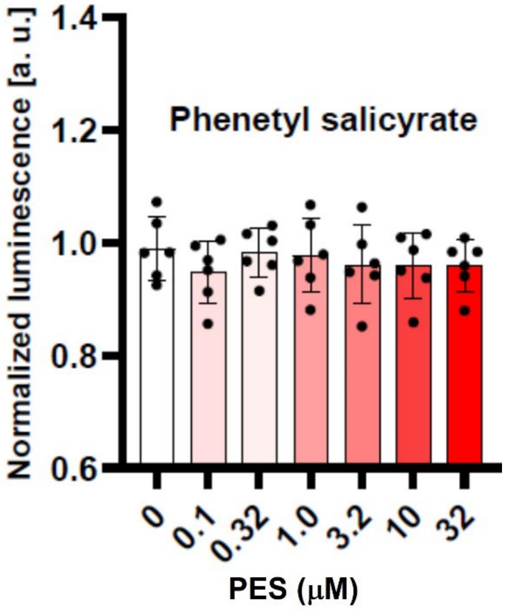

Figure S2 Effect of PES addition to the medium on cell proliferation

Cell toxicity of odorants exposed to the cells using CellTiter-Glo® 2.0 Luminescent Cell Viability Assay (Promega). The luminescence in each well was measured at 24 hours after changing medium supplemented with PES. The values of luminescence were normalized such that the value of each well without PES was defined 1.0. Error bar indicates s.d (n=8). Statistical analysis involved one-way ANOVA ($F(6, 35) = 0.416$, $p = 0.86$) followed by Dunnett's test.

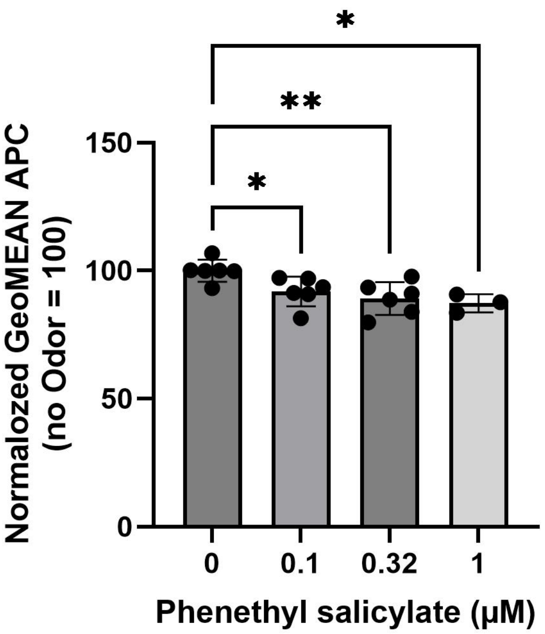

Figure S3 Effect of PES addition to the total expression of Or11g7

Total expression levels of Or11g7 after the addition of Phenyl salicylate (PES), The APC geometric mean values for Or11g7 without odor supplementation were normalized to 100. Error bars represent the standard deviation (SD). Statistical analysis was performed using one-way ANOVA followed by Dunnett's test ($p > 0.05$).

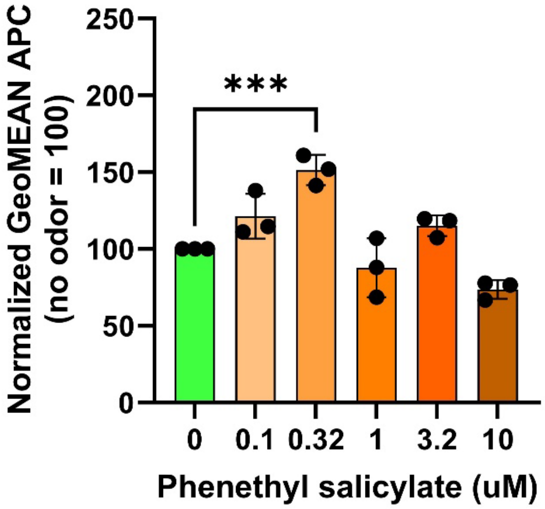

Figure S4 Effect of PES addition on Or11g7 cell surface expression when co-expressed with RTP1S.

All samples were co-transfected with mouse RTP1S and Or11g7. The APC geometric mean values for Or11g7 without PES supplementation were normalized to 100. Error bars represent the standard deviation (SD) for n=3. Statistical analysis was performed using one-way ANOVA followed by Dunnett's test (***$p < 0.001$).

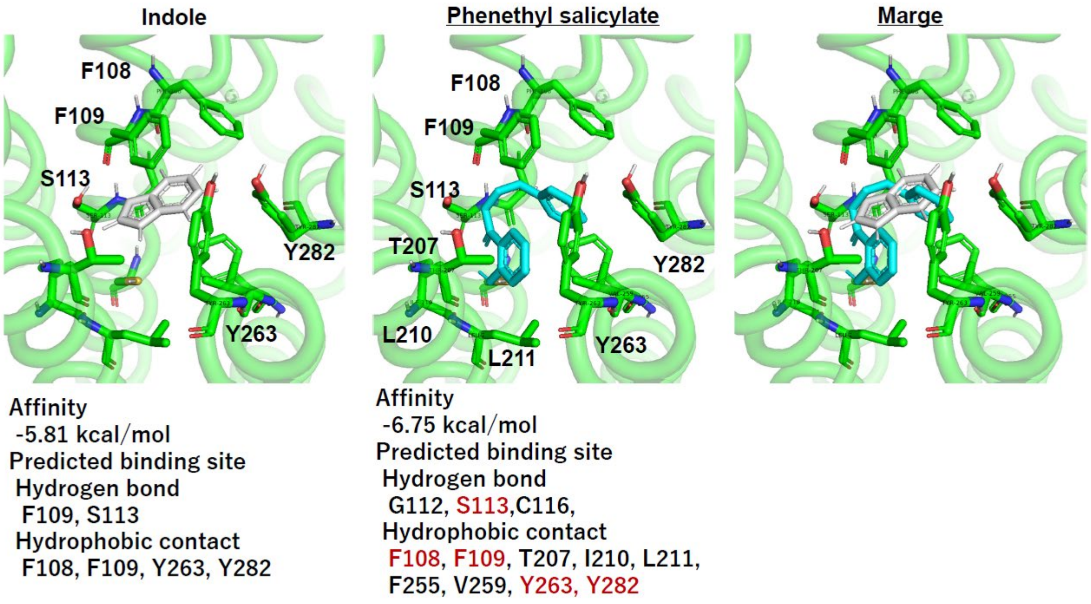

Figure S5 Docking simulation of Or11g7 with Indole and Phenethyl salicylate

Docking simulations were performed using AutoDock based on the structural model of Or11g7 generated with AlphaFold2.

Left) Binding with Indole, including binding energy and predicted interacting amino acids. Middle) Binding with Phenethyl salicylate. Right) Merged image of the two interactions.

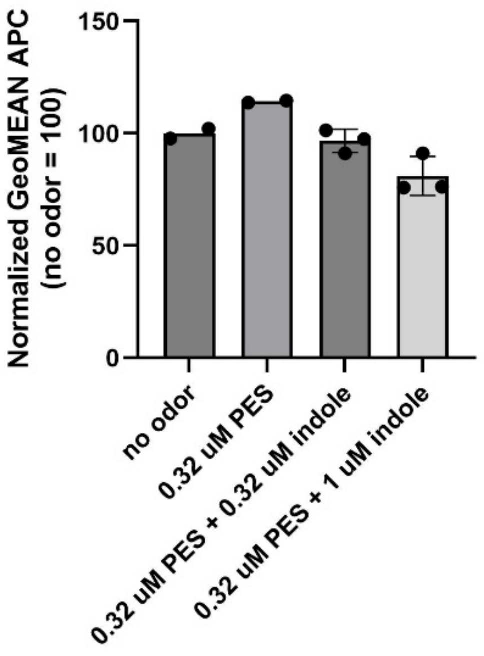

Figure S6 Competitive Inhibition of PES by Indole Addition

Cell surface expression levels of Or11g7 after the addition of Phenyl salicylate (PES) or Phenyl salicylate (PES) and Indole. The APC geometric mean values for Or11g7 without odor supplementation were normalized to 100. Error bars represent the standard deviation (SD).

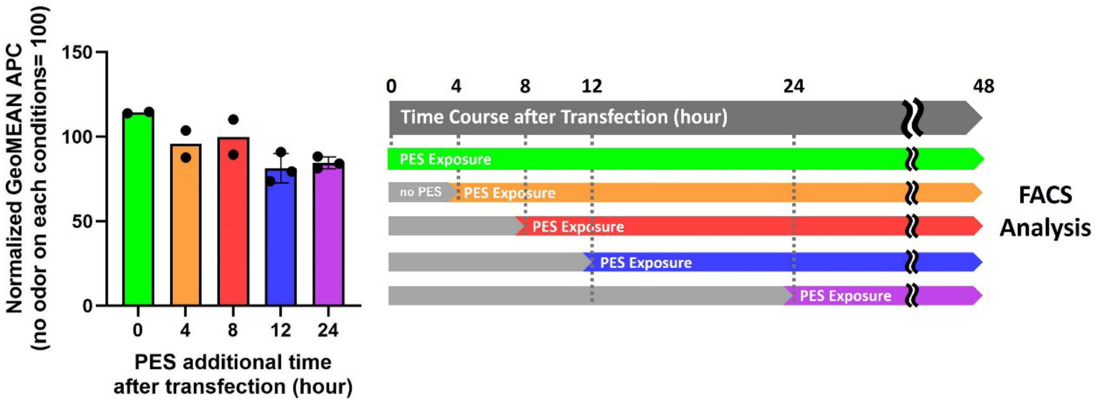

Figure S7 Kinetics effect of PES addition to the surface expression of Or11g7

Cell surface expression levels of Or11g7 after the addition of Phenyl salicylate (PES) at different time points post-transfection, with all expression levels analyzed 48 hours after transfection. The APC geometric mean values for Or11g7 without odor supplementation were normalized to 100. Error bars represent the standard deviation (SD).

Supplementary Method

Docking simulations

Docking simulations were performed using SeamDock available on the Pred-O3 website (https://odor.rpbs.univ-paris-diderot.fr/docking)[1, 2]. The structure of Or11g7 (UniProt ID: E9PV7) was modeled based on the AlphaFold2 predicted structure. The 3D molecular structures of the ligands (Indole and Phenethyl salicylate) were retrieved from PubChem. Box coordinates were set as follows: Center x: 0 Å, y: 0 Å, z: 0 Å, and Size x: 30 Å, y: 30 Å, z: 30 Å. The docking parameters were configured with the following settings: Software: AutoDock; Spacing: 0.375.

References

1. Achebouche, R.; Tromelin, A.; Audouze, K.; Taboureau, O., Application of artificial intelligence to decode the relationships between smell, olfactory receptors and small molecules. *Sci Rep* **2022,** 12, (1), 18817.

2. Murail, S.; de Vries, S. J.; Rey, J.; Moroy, G.; Tuffery, P., SeamDock: An Interactive and Collaborative Online Docking Resource to Assist Small Compound Molecular Docking. *Front Mol Biosci* **2021,** 8, 716466.
